# Correlations between Basal Trace Minerals and Hormones in Middle and Long-Distance High-Level Male Runners

**DOI:** 10.3390/ijerph17249473

**Published:** 2020-12-17

**Authors:** Javier Alves, Gema Barrientos, Víctor Toro, Francisco Javier Grijota, Diego Muñoz, Marcos Maynar

**Affiliations:** 1 Department of Sport Science, Faculty of Education, Pontifical University of Salamanca, C/Henry Collet, 52–70, CP, 37007 Salamanca, Spain; fjalvesva@upsa.es; 2 Department of Physiology, Faculty of Sports Science Faculty, University of Extremadura, University Avenue, s/n CP, 10003 Cáceres, Spain; vtororom@alumnos.unex.es (V.T.); fgrijota@nebrija.es (F.J.G.); diegomun@unex.es (D.M.); mmaynar@unex.es (M.M.)

**Keywords:** trace mineral, LH, testosterone, cortisol, insulin, runners

## Abstract

Several essential trace minerals play an important role in the endocrine system; however, toxic trace minerals have a disruptive effect. The aim of this research was to determine basal concentrations and the possible correlations between trace minerals in plasma and several plasma hormones in runners. Sixty high-level male endurance runners (21 ± 3 years; 1.77 ± 0.05 m; 64.97 ± 7.36 kg) participated in the present study. Plasma hormones were analyzed using an enzyme-linked immunosorbent assay (ELISA) and plasma trace minerals were analyzed with inductively coupled plasma mass spectrometry (ICP-MS). Correlations and simple linear regression were used to assess the association between trace minerals and hormones. Plasma testosterone concentrations were inversely correlated with manganese (r = −0.543; β = −0.410; *p* < 0.01), selenium (r = −0.292; β = −0.024; *p* < 0.05), vanadium (r = −0.406; β = −1.278; *p* < 0.01), arsenic (r = −0.336; β = −0.142; *p* < 0.05), and lead (r = −0.385; β = −0.418; *p* < 0.01). Plasma luteinizing hormone (LH) levels were positively correlated with arsenic (r = 0.298; β = 0.327; *p* < 0.05) and cesium (r = 0.305; β = 2.272; *p* < 0.05), and negatively correlated with vanadium (r = −0.303; β = −2.467; *p* < 0.05). Moreover, cortisol concentrations showed significant positive correlations with cadmium (r = 0.291; β = 209.01; *p* < 0.05). Finally, insulin concentrations were inversely related to vanadium (r = −0.359; β = −3.982; *p* < 0.05). In conclusion, endurance runners living in areas with high environmental levels of toxic minerals should check their concentrations of anabolic hormones.

## 1. Introduction

Endurance runners perform high amounts of training with long aerobic exercise sessions, where they run for a long time at intensities lower than the second ventilatory threshold (VT_2_); training that causes great physiological stress and induces changes and adaptations in their gonadal and cortical axis [1]. Hackney [2] reported a dysfunction in the hypothalamus-hypophyseal-testicular axis, defined as “exercise-hypogonadal male condition”, which leads to low levels of chronic basal testosterone (T) in endurance athletes [3], without changes in luteinizing hormone concentrations (LH), which may indicate a testicular dysfunction [4]. Other authors, however, found normal concentrations of T and LH in endurance athletes [5,6].

Trace minerals (TM) are present in body tissues as an essential part of many physiological functions, and their deficiency or excess can lead to metabolic disorders [7]. Some of these minerals are essential for human health although they may be harmful in high concentrations; other minerals to which we are exposed are considered toxic for health [8]. The main exposure sources to TM is diet and air [8]. Significant interactions have been observed between TM levels and the endocrine system since TM influence the metabolism of hormones and vice versa, so changes in hormone concentrations could affect the metabolism and redistribution of TM [9].

Essential TM such as copper (Cu), selenium (Se), and zinc (Zn) have been found to play a fundamental role in the regulation of testicular function, in the spermatogenesis process, and the production of androgens [7]. Moreover, an inverse correlation has been reported between molybdenum (Mo) and T in subjects with Zn deficiency [10] and in animals an excess of manganese (Mn) concentrations has been found to negatively impact the function of Leydig cells [11].

On the other hand, toxic minerals are considered disruptors of hormonal metabolism; the adverse effects of arsenic (As), cadmium (Cd), lead (Pb), and mercury (Hg) on testicular function have been widely reported to interfere in T levels [12,13].

Cortisol (C) is a catabolic hormone regulated by the hypothalamus-hypophysis-adrenal axis that facilitates the mobilization of substrates during exercise to improve athletic performance [14], and its concentrations can be kept high up to 48 h after exhaustive long-term exercise, interfering with the recovery process [1]. Few studies relating TM with this hormone were found; Soria et al. found that increases in C concentrations in trained athletes positively correlated with increases in Zn and Se levels, which suggests that it occurs because of the participation of these elements in the prevention of oxidative stress [15].

Insulin (I) is considered an anabolic hormone related to blood glucose control, energy balance, and the metabolism of carbohydrate, fat, and protein [16]. A decrease in plasma I concentrations has been observed during exercise, followed by an increase in I synthesis at the end of exercise, to promote glycogen repletion through lower glucose production and utilization and an increase in fat oxidation [17]. Insulin and its relationship with TM have been widely studied in metabolic diseases such as diabetes; for example, by Soria et al. [15], although this study found no correlation between I and initial plasma concentrations of Se, Zn, Mn, and cobalt (Co) in trained triathletes. In another study, Tubek [18] reported a positive correlation with Zn, which would support the hypothesis that this mineral is necessary for the synthesis, accumulation, and release of insulin [19].

Research has reported on the relationship between energy intake and the synthesis of anabolic hormones [20,21]. High-level endurance runners in training periods with an intake below 45 calories per day per kg of fat-free mass (FFM) had decreases in LH and T hormones [22]. As mentioned above, significant interactions have been observed between TM concentrations and the endocrine system. It is known that TM have biological implications for endocrine processes [23], and a large number of studies examine these relationships in animals or healthy subjects; however, few studies have examined the relationship between hormones and TM in athletes [9,15].

Previous studies by our research group reported significant differences in plasma TM concentrations between sedentary subjects and endurance runners. Runners had higher plasma concentrations of Mn, Mo, chrome (Cr), nickel (Ni), and rubidium (Rb) and lower concentrations of Se and Zn [24,25,26]. Higher plasma concentrations of toxic TM, such as beryllium (Be), cesium (Cs), Pb, and Cd, were also found in runners [27].

Therefore, the objectives of the study were (i) to check basal concentrations of hormones (testosterone, LH, cortisol, and insulin) and TM in highly trained endurance athletes, and (ii) to study the possible relationships between trace minerals and hormone metabolism, since hormones play a fundamental role in runners’ energy metabolism, recovery, and performance.

## 2. Materials and Methods

### 2.1. Participants

Sixty male endurance runners (21 ± 3 years old; height 1.77 ± 0.05 m) took part in the study. They had a personal best in modalities of 3:37.79–4:08.24 for 1500 m and 13:11.01 and 15:16.78 for 5000 m.

The criteria for the selection of participants in our study were: to be runners who had been living in the same region for at least three years, at a latitude of 39°28′35.36″ N, to have trained regularly and six days a week during the previous six years, and to have participated in national and international track and field and cross-country competitions in that period. None of the subjects had taken regular medication, anti-inflammatory medications, or TM supplementation for 3 months prior to the study. All runners were previously informed about the purpose of the study and signed their voluntary informed consent. This research was conducted under the Helsinki Declaration ethical guidelines, updated at the World Medical Assembly in Fortaleza in 2013 for research with human participants, and the protocol was approved by the Ethics Committee of the University of Extremadura (52/2012).

The participants’ characteristics are summarized in Table 1.

### 2.2. Nutritional Evaluation

The runners were following a similar diet. All subjects were trained to complete a nutritional questionnaire, on two pre-assigned weekdays and one weekend day. Runners indicated quantity (in grams) of every food consumed. Then, their dietary intakes were analyzed using different food composition tables [8,28,29].

### 2.3. Training Characteristics

Runners’ training routines were monitored (some runners did double sessions), performing an average distance between 80 and 155 km per week during the sport season (75–85% aerobic running and 15–25% high intensity running). The kilometers that runners trained with an intensity higher than VT_2_ according to the three-phase model [30] were identified as high intensity. Additionally, they trained one-two weekly sessions of resistance training depending on the period of the season.

### 2.4. Anthropometric Measurements

The anthropometric measurements were obtained at the same time (09:00–10:00 a.m.) in equal conditions and were carried out by an accredited operator in kinanthropometric techniques Level 1. Runners did not perform intense training 72 h before the sampling. Fat mass and fat-free mass content were calculated from the sum of 6 skinfolds (∑6) (abdominal, tricipital, suprailiac, subscapularis, thigh, and calf), in accordance with the International Society for the Advancement of Kinanthropometry recommendations [31]. Skinfold thicknesses were measured with a Harpenden caliper (Holtain Skinfold Caliper, Crosswell, Crymych, UK). Body fat percentage was calculated according to Jackson and Pollock [32]. Body weight was measured to the nearest 0.01 kg using a digital scale (Seca 769, Hamburg, Germany) and body height was measured to the nearest 0.1 cm using a wall-mounted stadiometer (Seca©, Hamburg, Germany).

### 2.5. Sample Collection

After the anthropometric measurements, 10 mL of venous blood was drawn from each runner. Then, the samples were collected into tubes (previously washed with diluted nitric acid) with ethylenediaminetetraacetic acid.

Later, samples were centrifuged to isolate the plasma which was deposited into an Eppendorf tube (previously washed with diluted nitric acid) and conserved at −80 °C until biochemical analysis.

### 2.6. Sample Determination

In order to determine the concentration of trace minerals, analyses were performed using inductively coupled plasma mass spectrometry (ICP-MS) following the method described by Maynar et al. [24].

The decomposition of the organic matrix was achieved by heating it for 10 h at 90 °C after adding 0.8 mL HNO_3_ and 0.4 mL H_2_O_2_ to 2 mL of plasma samples. The samples were then dried at 200 °C on a hot plate. Sample reconstitution was carried out by adding 0.5 mL of nitric acid, 10 μL of indium (In) (10 mg/L) as an internal standard, and ultrapure water to complete 10 mL. Digested solutions were analyzed with an ICP-MS Nexion model 300D (PerkinElmer, Inc., Shelton, CT, USA). Three replicates were analyzed per sample. The values of the standard materials of each element (10 μg/L) used for quality controls were in accordance with intra and inter-assay coefficient variations of less than 5%.

The hormone quantification was conducted using enzyme-linked immunosorbent assay (ELISA) with an ER-500 (Sinnowa, Germany), using the commercial tests for insulin, cortisol, testosterone, and luteinizing hormone from CisRadioquímica, SA (Madrid, Spain). Hormonal analyses were performed in duplicate by the same technician. Coefficients of variation (between and within) were less than 10% for all biochemical analyses.

### 2.7. Statistical Evaluations

Statistical analyses were performed with IBM SPSS 21 for Windows (IBM Co., Armonk, NY, USA). The results are expressed as x ± s, where “x” represents mean values and “s” the standard deviation. A Kolmogorov–Smirnov test was used to analyze the normality of the distribution of variables, and the homogeneity of the variances was analyzed with the Levene’s test. A simple linear regression model was used to determine associations between TM concentrations and plasma hormone concentrations. Pearson’s correlation coefficient (r), the β coefficients, and determination coefficients (R^2^) were calculated. Strength of linear association was established according to Chan [33]. A *p* < 0.05 was considered statistically significant.

## 3. Results

Table 2 presents data on the nutritional intake of all athletes per day.

Nutritional intakes were adequate for the athletic performance of the runners in our study [34]. Energy availability (EA) is defined as dietary energy intake minus exercise energy expenditure/FFM. For healthy young adults, an appropriate energy balance is associated with ≥45 calories per day per kg of FFM [21].

Table 3 and Table 4 show the plasma values of trace minerals and plasma hormones, respectively. LH (7.94 ± 2.95 mIU/mL), insulin (7.31 ± 4.03 mIU/mL), testosterone (6.52 ± 1.14 ng/mL), and cortisol (95.81 ± 33.85 ng/mL) for all runners participating in the study.

The correlation coefficients and simple linear regressions are shown in Table 5.

Plasma LH levels were positively correlated with As (r = 0.298; β = 0.327; *p* < 0.05) and cesium (Cs) (r = 0.305; β = 2.272; *p* < 0.05), and negatively related with V (r = −0.303; β = −2.467; *p* < 0.05). Finally, insulin concentrations were inversely correlated to V (r = −0.359; β = −3.982; *p* < 0.05).

Plasma testosterone concentrations were inversely associated with Mn (r = −0.543; β = −0.410; *p* < 0.01), Se (r = −0.292; β = −0.024; *p* < 0.05), vanadium (V) (r = −0.406; β = −1.278; *p* < 0.01), As (r = −0.336; β = −0.142; *p* < 0.05), and Pb (r = −0.385; β = −0.418; *p* < 0.01). On the other hand, cortisol levels showed significant positive correlations with Cd (r = 0.291; β = 209.01; *p* < 0.05).

## 4. Discussion

In our runners, energy availability, macronutrients, and TM intake from the diet were appropriate according to the recommended intakes [8,35].

Plasma Co concentrations were higher, although without negative health effects in healthy individuals [36].

Runners’ plasma LH and T hormones were within normal ranges [37], although plasma T concentrations were lower than those reported in untrained males [38].

An inverse correlation was found between plasma concentration of LH and V plasma concentrations. Vanadium is considered a toxic mineral [39]. However, there are still some ambiguous assertions about its possible essentiality that require further studies in the future [40]. The correlations found in our study could be related to previous studies in rats suggesting that the nucleus of Leydig cells are a possible target of V, and its excessive accumulation could produce a transient suppression in testicular function by reducing plasma concentrations of LH and T [41]. This would be a consequence of an increase in oxidative stress in the testes as demonstrated by Chandra et al. [42], where rats receiving injections of sodium vanadate had high levels of indices of lipid peroxidation in testicular tissues compared to control tissues, caused by a decrease in the activity of antioxidant enzymes. It is known that physical training in high-level runners increases reactive oxygen species (ROS) production and the possibility of oxidative stress (OS) [43]. Therefore, the runners’ antioxidant activity would not be sufficient to compensate for the production of ROS by V and accumulation of training loads.

Plasma concentration of LH had a positive correlation with plasma As concentration. Banerjee et al. [44] found that decreases in serum T concentrations were observed in animals with As poisoning. In males, As may induce gonad dysfunction through declined T synthesis [45]. In another study, Zeng et al. [46] concluded that As has an inhibitory effect on the production of T. These data could be related to the correlations found in our athletes between plasma As concentration and the plasma concentration of LH and serum T. Athletes’ plasma As concentrations were within reasonable limits, and according to these correlations, we could infer that the increase in As concentration would lead to a decrease in T concentrations and this would increase plasma concentrations of LH to try to compensate for the reduction of T and increase its synthesis. More research studies on humans are needed.

A correlation was also found between LH and Cs. Previous studies have reported that chronic Cs exposure does not negatively affect steroid hormone concentrations or the correct functioning of the hypothalamus-hypophysis-testicular (HPT) axis [47]; however, with our current knowledge we cannot explain this relationship.

A negative correlation was found between Mn and total plasma T. Mn is an essential mineral in specific physiological processes, and is a compound of manganese superoxide dismutase (Mn-SOD) enzyme which neutralizes superoxide radicals during physical exercise [48]. In their study, Plumlee and Ziegler [49] indicated that a deficiency of this mineral can lead to testicular and skeletal dysfunctions, which could be linked to the dependence of testicular Leydig cells on plasma Mn for the testicular synthesis of this hormone. Lee et al. [50] showed that Mn acts on the hypothalamus inducing the secretion of LH, a hormone that controls the production of T from Leydig cells, but in this study, there was no correlation with LH although there was a correlation with T.

Another interesting positive correlation was T with plasma Se concentrations. This correlation in our athletes could be related to what was described earlier; Se would be necessary for the healthy metabolism of T and normal testicular morphology, and this could explain the presence of several selenium-proteins in male gonads [51,52]. Furthermore, Pond et al. [53] seem to indicate that alterations in spermatogenesis could be due to lack of activity of the glutathione peroxidase (GPx) enzymes. In a recent review, it was concluded that essential hormonal regulators and testicular functions can be negatively affected when there is an uncontrolled generation of ROS with respect to the antioxidant defense mechanism, as a consequence of lack of activity of the GPx enzymes, which would alter the production of T [54]. Therefore, it is essential for runners to maintain adequate Se concentrations to ensure correct activity of antioxidant enzymes as well as the synthesis of T.

A negative correlation was found between plasma T and V concentrations. As previously discussed in plasma LH concentrations, V has been reported to increase ROS generation in the body, which would induce oxidative stress, and could reduce testicular T synthesis [42]. Recently, Zwolak [55] reported the positive role of several dietary antioxidants in vanadium toxicology.

A negative correlation with As was also found with plasma T. Arsenic is a toxic TM that due to its ability to generate ROS could alter the synthesis of T [45], a relationship that was previously discussed.

Testosterone was also found to have an important negative correlation with Pb. The toxic effects of Pb have been reported to have an adverse impact on the central nervous system, liver, kidneys, and the reproductive system [56]. This correlation in our athletes could indicate that lowering serum concentrations of Pb produced an increase in T concentrations. It has been reported that Pb causes oxidative stress by generating ROS that results in critical damage to several biomolecules such as DNA, enzymes, proteins, and the membrane of lipids, as well as a decrease in antioxidant activity [57]. Increases in ROS production due to training and the toxic action of this mineral could affect the synthesis of T in runners. Darbandi et al. [54] reported the excess generation of ROS with respect to the antioxidant defense mechanism, which could alter the synthesis of T in endurance runners.

In relation to C, runners’ baseline plasma concentrations were within reference ranges [58]. Cortisol increases its synthesis during training and can remain elevated up to 48 h after finishing [59]; however, our runners did not perform intense training 72 h before the sampling. We only found a positive correlation with Cd. Cadmium is an environmental toxin and an endocrine disruptor, and humans are very sensitive to its toxic effects. Recent data have shown that exposure to Cd leads to an increase in plasma levels of adrenocorticotropic hormone (ACTH) [60], a hormone secreted by the pituitary gland that controls the release of C in the adrenal gland [61]. Pérez-Cadahía et al. [62] reported positive relationships between C and Cd, that indicate that this mineral could have an implication with the catabolic processes that occur in the body, so the runner’s body attempts to eliminate Cd although its concentrations are normal in order to avoid this negative effect [63]. Future studies are required to clarify this point.

With respect to I, baseline plasma concentrations of the runners were within normal ranges [58]. A negative correlation was found with plasma V concentration. Therefore, this could indicate the need for this mineral to synthetize I or for the correct functioning of this hormone. In this respect, Cam et al. [64] concluded that V enhances the effects of endogenous circulating I, and even could be used as an I supplement to avoid the insulin resistance that its exogenous chronic administration can cause [65]. More studies are needed to establish the long-term effects of V treatment and to establish doses in different groups of people [66].

The limitations of this study include that the intake of TM by the runners was obtained using a self-report questionnaire that could introduce some inaccuracies. Another limitation was that some of our assessments may be somewhat speculative and may not allow us to have a solid discussion, due to the scarcity of studies in runners.

## 5. Conclusions

Plasma hormone concentrations and several trace mineral concentrations in high training endurance runners were in normal ranges.

LH correlated with the plasma concentrations of vanadium, arsenic, and cesium. Testosterone reflected correlations with manganese, selenium, arsenic, lead, and vanadium concentrations. Cortisol showed a correlation with cadmium concentration, and insulin correlated with vanadium concentration.

Essential and toxic trace elements are involved in the endocrine system, and their deficiency or excess could lead to metabolic disorders in endurance runners. Our results indicate that endurance athletes living and training in places with high environmental levels of toxic minerals should check concentrations of anabolic hormones (LH and T) as they could suffer decreases in their plasma concentrations that would negatively affect sports performance.

## Figures and Tables

**Table 1:** Anthropometric and body composition values in the runners.

Parameters	Runners	Ranges
Height (m)	1.77 ± 0.05	1.55–1.88
Body mass (kg)	64.97 ± 7.36	49.7–78.1
Fat mass (kg)	5.35 ± 1.01	3.45–7.87
Fat mass (%)	8.20 ± 1.06	7.12–1.06
Fat-free mass (FFM) (kg)	59.77 ± 6.57	42.13–71.64
∑6 skinfold (mm)	45.61 ± 9.71	35.98–53.21

**Table 2:** Energy, macronutrients, and trace minerals in the runners.

Parameters, Recommended Intake	Intake
Energy (kcal/d)	2885.62 ± 649.28
HC (g/kg/d)	5.70 ± 1.31
Proteins (g/kg/d)	1.79 ± 1.57
Lipids (g/kg/d)	1.67 ± 1.48
EA (kcal/kg FFM/day)	46.63 ± 4.49
Arsenic (12–300 mg/d)	16.92 ± 80.20
Boron (0.75–1.35 mg/d)	1.34 ± 1.48
Beryllium (<50 µg/d)	9.71 ± 9.02
Cadmium (<70 µg/d)	23.33 ± 15.39
Cobalt (200–300 µg/d)	295.94 ± 215.07
Copper (2000–3000 µg/d)	1676.06 ± 566.89
Lithium (180–550 µg/d)	367.03 ± 396.86
Manganese (2500–5000 µg/d)	3378.13 ± 1440.05
Molybdenum (75–400 µg/d)	309.02 ± 182.03
Lead (<400 µg/d)	209.48 ± 141.81
Rubidium (1.5–7 mg/d)	3.896 ± 4.712
Selenium (50–200 µg/d)	76.54 ± 44.96
Strontium (1000–2300 µg/d)	1889.02 ± 1782.25
Vanadium (10–70 µg/d)	25.52 ± 29.31
Zinc (10–15 mg/d)	11.12 ± 3.68

**Table 3:** Trace mineral concentrations in the runners.

Trace Minerals	Runners	Range
Arsenic (µg/L)	2.35 ± 2.70	0.23–12.00
Boron (µg/L)	8.63 ± 10.96	0–60.58
Beryllium (µg/L))	0.07 ± 0.03	0–0.14
Cadmium (µg/L)	0.07 ± 0.05	0.01–0.23
Cesium (µg/L)	0.69 ± 0.40	0.31–1.87
Cobalt (µg/L)	0.68 ± 0.10	0.47–0.88
Copper (µg/L)	693.16 ± 132.53	454.9–937.01
Lithium (µg/L)	1.38 ± 0.79	0.36–4.72
Manganese (µg/L)	2.06 ± 1.49	0.19–5.46
Molybdenum (µg/L)	0.62 ± 0.59	0.1–3.33
Lead (µg/L)	0.96 ± 1.07	0.01–4.94
Rubidium (µg/L)	138.49 ± 22.98	98.8–185.98
Selenium (µg/L)	96.5 ± 13.8	69.7–124.1
Strontium (µg/L)	26.24 ± 8.12	14.83–47.47
Vanadium (µg/L)	0.29 ± 0.37	0–1.78
Zinc (µg/L)	792.20 ± 143.91	539.52–1210.95

**Table 4:** Plasma hormonal concentrations in runners.

Hormones	Runners	Ranges
LH (mIU/mL)	7.94 ± 2.95	2.94–16.03
Insulin (µIU/mL)	7.31 ± 4.03	1.39–18.91
Testosterone (ng/mL)	6.52 ± 1.14	4.13–9.94
Cortisol (ng/mL)	95.81 ± 33.85	42.40–170.92

**Table 5:** Correlations and simple linear regressions between trace minerals and hormones.

	LH (mIU/mL)				Insulin (µIU/mL)				Testosterone (ng/mL)				Cortisol (ng/mL)			
	r	β (95% CI)	R^2^	*p*	r	β (95% CI)	R^2^	*p*	r	β (95% CI)	R^2^	*p*	r	β (95% CI)	R^2^	*p*
As	0.298	0.327 (0.01/0.63)	0.089	0.040	−0.015			0.917	−0.336	−0.142 (−0.26/−0.02)	0.113	0.020	−0.082			0.580
B	−0.142			0.335	0.007			0.963	−0.105			0.476	−0.084			0.571
Be	−0.075			0.613	0.237			0.104	0.080			0.589	−0.266			0.068
Cd	0.095			0.521	−0.135			0.362	−0.001			0.996	0.291	209.01 (4.90/413.1)	0.085	0.045
Cs	0.305	2.272 (0.16/4.38)	0.093	0.035	0.186			0.205	−0.234			0.110	−0.073			0.622
Cu	−0.099			0.502	0.020			0.894	−0.204			0.163	−0.149			0.312
Li	−0.251			0.085	−0.042			0.776	0.053			0.720	0.135			0.360
Mn	−0.226			0.122	−0.197			0.180	−0.543	−0.410 (−0.59/−0.22)	0.295	0.000	−0.268			0.066
Mo	−0.158			0.285	0.160			0.278	0.209			0.154	0.102			0.492
Pb	−0.045			0.759	−0.220			0.133	−0.385	−0.418 (−0.71/−0.12)	0.148	0.007	−0.283			0.052
Rb	−0.144			0.032	0.195			0.185	0.033			0.826	0.098			0.508
Se	0.043			0.773	0.027			0.855	−0.292	−0.024 (−0.04/−0.01)	0.085	0.044	−0.190			0.195
Sr	−0.131			0.376	−0.223			0.127	−0.229			0.117	−0.196			0.183
V	−0.303	−2.467 (−4.71/−0.16)	0.092	0.036	−0.359	−3.982 (−7.05/−0.90)	0.129	0.012	−0.406	−1.278 (−2.13/−0.425)	0.165	0.004	−0.212			0.148
Zn	0.276			0.058	0.102			0.491	0.135			0.361	0.003			0.981

LH: luteinizing hormone; As: arsenic; B: boron; Be: beryllium; Cd: cadmium; Cs: cesium; Cu: copper; Li: lithium; Mn: manganese; Mo: molybdenum; Pb: lead; Rb: rubidium; Se: selenium; Sr: strontium; V: vanadium; Zn: zinc; r: Pearson’s coefficient of correlation; β: beta coefficient; CI: confidence interval; R^2^: coefficient of determination; *p*: *p*-value.
