# Erratum: Alves, J., et al. Correlations between Basal Trace Minerals and Hormones in Middle and Long-Distance High-Level Male Runners. *International Journal of Environmental Research and Public Health* 2020, *17*, 9473

**DOI:** 10.3390/ijerph18052289

**Published:** 2021-02-25

**Authors:** Javier Alves, Gema Barrientos, Víctor Toro, Francisco Javier Grijota, Diego Muñoz, Marcos Maynar

**Affiliations:** 1 Department of Sport Science, Faculty of Education, Pontifical University of Salamanca, C/Henry Collet, 52–70, CP, 37007 Salamanca, Spain; fjalvesva@upsa.es; 2 Department of Physiology, Faculty of Sports Science Faculty, University of Extremadura, University Avenue, s/n CP, 10003 Cáceres, Spain; vtororom@alumnos.unex.es (V.T.); diegomun@unex.es (D.M.); mmaynar@unex.es (M.M.); 3 Department of Education, Faculty of Language and Education, Antonio de Nebrija University, C/Del Hostal, 28248 Hoyo de Manzanares, Madrid, Spain; fgrijota@nebrija.es

The authors wish to correct the following erratum in this paper [1].

The fourth author´s affiliation is not correct. It should read:

1. Department of Sport Science, Faculty of Education, Pontifical University of Salamanca, C/Henry Collet, 52–70, CP, 37007 Salamanca, Spain; fjalvesva@upsa.es (J.A.)
2. Department of Physiology, Faculty of Sports Science Faculty, University of Extremadura, University Avenue, s/n CP, 10003 Cáceres, Spain; vtororom@alumnos.unex.es (V.T.); diegomun@unex.es (D.M.); mmaynar@unex.es (M.M.)
3. Department of Education, Faculty of Language and Education. Antonio de Nebrija University, C/Del Hostal, 28248 Hoyo de Manzanares, Madrid, Spain; fgrijota@nebrija.es (F.J.G.)

## Data Availability

Data supporting reported results can be found in the next link. https://www.mdpi.com/1660-4601/17/24/9473.
